# Is abdominal wall contraction important for normal voiding in the female rat?

**DOI:** 10.1186/1471-2490-7-5

**Published:** 2007-03-07

**Authors:** Phillip P Smith, Christopher P Smith, Timothy B Boone, George T Somogyi

**Affiliations:** 1 Scott Department of Urology, Baylor College of Medicine, Houston TX USA

## Abstract

**Background:**

Normal voiding behavior in urethane-anesthetized rats includes contraction of the abdominal wall striated muscle, similar to the visceromotor response (VMR) to noxious bladder distension. Normal rat voiding requires pulsatile release of urine from a pressurized bladder. The abdominal wall contraction accompanying urine flow may provide a necessary pressure increment for normal efficient pulsatile voiding. This study aimed to evaluate the occurrence and necessity of the voiding-associated abdominal wall activity in urethane-anesthetized female rats

**Methods:**

A free-voiding model was designed to allow assessment of abdominal wall activity during voiding resulting from physiologic bladder filling, in the absence of bladder or urethral instrumentation. Physiologic diuresis was promoted by rapid intravascular hydration. Intercontraction interval (ICI), voided volumes and EMG activity of the rectus abdominis were quantified. The contribution of abdominal wall contraction to voiding was eliminated in a second group of rats by injecting botulinum-A (BTX, 5 U) into each rectus abdominis to induce local paralysis. Uroflow parameters were compared between intact free-voiding and BTX-prepared animals.

**Results:**

Abdominal wall response is present in free voiding. BTX preparation eliminated the voiding-associated EMG activity. Average per-void volume decreased from 1.8 ml to 1.1 ml (p < 0.05), and reduced average flow from 0.17 ml/sec to 0.11 ml/sec (p < 0.05). Intercontraction interval (ICI) was not changed by BTX pretreatment.

**Conclusion:**

The voiding-associated abdominal wall response is a necessary component of normal voiding in urethane anesthetized female rats. As the proximal urethra may be the origin of the afferent signaling which results in the abdominal wall response, the importance of the bladder pressure increment due to this response may be in maintaining a normal duration intermittent pulsatile high frequency oscillatory (IPHFO)/flow phase and thus efficient voiding. We propose the term Voiding-associated Abdominal Response (VAR) for the physiologic voiding-associated EMG/abdominal wall response, to distinguish it from the visceromotor response (VMR) to noxious bladder distension.

## Background

Electromyographic (EMG) evidence of abdominal wall contraction has been observed to accompany voiding in awake rats during natural voiding and suprapubic cystometry [1] as well as in urethane-anesthetized rats during transurethral cystometry [2]. Similar contraction of the abdominal wall occurs in response to noxious distension of the bladder and bowel in mice and rats, and is termed the visceromotor reflex (VMR) [3]. Whether the physiologic voiding-associated abdominal wall activity is the same reflex as the nociceptive VMR, and if it is necessary for normal voiding in rats has not been reported.

Normal voiding in the rat has been divided into four phases [4]. Upon the signal to void, the bladder first pressurizes as a result of detrusor contraction; continued tonic rhabdosphincter activity prevents flow [5]. At threshold bladder pressure, the second phase begins and the external rhabdosphincter (EUS) relaxes and contracts in a pulsatile fashion, which results in a rhythmic oscillation of bladder pressure matched by a complementary oscillation of urethral EUS pressure. These oscillations are termed intermittent pulsatile high frequency oscillations (IPHFOs), and it is during IPHFO phase that flow occurs [6]. The third phase is marked by a rebound increase in bladder pressure following termination of the IPHFOs, and is followed by the final phase, resolution of the bladder contraction. Elimination of the rhabdosphincter function via neuromuscular blockade has been reported to impair voiding efficiency [7], therefore an element of urethral resistance to flow appears to be necessary for normal efficient voiding in the rat.

Rat voiding may thus be considered as a pressurized vessel releasing its contents against an intermittently relaxing resistance. Abdominal pressurization caused by the voiding-associated abdominal wall contraction may be required in this process. We therefore hypothesized that the abdominal wall activity observed during physiologic voiding is a necessary component of normal voiding in the rat.

Cystometric study either via transurethral or suprapubic catheter potentially alters the micturition reflex by direct stimulation of bladder or urethral afferents by the catheter or its placement, by supraphysiologic bladder filling rates, or by urothelial stimulation by non-urine infusion fluids. Our aim was to investigate the occurrence and necessity of voiding-associated abdominal wall activity during physiologic voiding in anesthetized rats. First, we proposed to design a free voiding model which would allow quantification of the abdominal wall EMG response and uroflow parameters during physiologic voiding in urethane-anesthetized rats with an uninstrumented lower urinary tract. Second, the necessity of the voiding-associated abdominal wall response for a normal uroflow response would be assessed by selective paralysis of the abdominal wall using botulinum toxin-A (BTX), thus removing the abdominal pressure contribution to voiding.

## Methods

A total of 15 urethane-anesthetized (1.2 gm/kg) female SD rats weighing 250–300 g were used for this study. Institutional Animal Care and Use Committee guidelines were observed. Physiologic data were collected, recorded, and analyzed using WinDaq software (DataQ software, Dayton Ohio) and Microsoft Excel.

All animals underwent a "free voiding" study, designed to allow quantification of the voiding response resulting from bladder filling from physiologic diuresis without instrumentation of the lower urinary tract. Vascular access for infusion and blood pressure measurement was obtained via a polyethylene catheter (PE50) placed into a femoral artery after anesthetization. EMG electrodes were placed into the right rectus abdominis, 1 cm apart longitudinally, via a small midline lower abdominal skin incision. The insulated portion of each electrode was sutured to the skin and the incision was closed. A second set of electrodes was placed into the superficial peri-urethral perineal musculature via small skin incisions, one on each side of the urethra, and secured with sutures. The animal was then placed into prone position with the urethral meatus over a 10 ml collection cup attached to a force transducer to quantify voided volume. The femoral artery catheter was connected to a Harvard PHD2000 infusion pump, with an in-line pressure transducer. The animals were left in this position until several voids had occurred. Following an initial 2 ml bolus delivered over 1 minute, animals were infused with normal saline via the arterial line at a rate of 0.08 ml/minute. This infusion rate was selected in order to produce a maximal physiologic diuresis in response to subacute volume loading [8]. The animal and transducers were contained within a large galvanic cage sharing electrical ground with the amplifier cabinet. The EMG signals were not filtered, and were differentially amplified by 1000 using Grass P511 amplifiers. EMG activity of the rectus abdominis and superficial perineal muscles, blood pressure, and voided volume were continuously recorded. Animals were euthanized with carbon dioxide at the conclusion of the experiment.

Seven of the free voiding rats were infused with normal saline via the arterial line (FV group). In eight other rats, the rectus abdominis muscle was injected with BTX (10 U), 48 hours prior to the free voiding study. Under isofluorane (2%) inhalation anesthesia, a midline skin incision was made and the skin reflected off of the underlying muscles. The toxin was injected using five evenly spaced injections into the length of each muscle belly. The skin was closed with continuous suture and the animal allowed to recover. Ampicillin 150 mg/kg intramuscular was administered prior to surgery.

**Figure 1:**
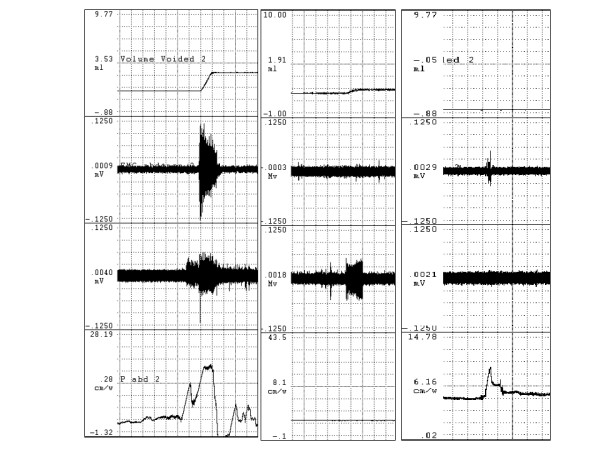
**Voiding-associated abdominal and perineal responses**. Tracings demonstrating voiding-associated abdominal and perineal responses, by EMG and by intraperitoneal catheter. Top tracing is voided volume, the slope of the volume change is the urine flow rate. The middle two tracings are rectus abdominis and perineal EMG tracings, respectively. The bottom tracing is intra-abdominal pressure. The left column is voiding-associated response in intact rat. The middle column demonstrates the lack of abdominal wall EMG and pressurization response in a botulinum-toxin prepared rat. The right column demonstrates pressure deflection associated with manually squeezing the abdomen in the absence of voiding.

To confirm that the voiding-associated abdominal wall EMG response correlated with a rise in intra-abdominal pressure and that loss of this increased intensity signified a loss of voiding-associated intra-abdominal pressurization, a pressure catheter was placed intraperitoneally in one intact free voiding rat and two of the BTX group, using a 20-gauge angiocath inserted into the left abdominal flank. A fluid column was established by injecting two to three milliliters of saline, and a pressure transducer was connected to monitor intra-abdominal pressure. Position and function was checked by manually squeezing the abdomen to produce a positive pressure deflection. An example of the pressure tracing obtained in response to voiding and to abdominal squeeze is demonstrated in Figure 1. This method gave a reasonably stable pressure tracing lasting one-two hours without degradation. Reinforcement of the fluid column with a small (<1 ml) bolus of saline via the intraperitoneal catheter corrected this degradation in the BTX animals. This problem was eliminated in the intact rat by using a syringe pump to administer a 0.050 ml/minute infusion through the abdominal pressure catheter (this animal was not included in the data analysis due to the difference in infusion method).

Voiding and EMG parameters were analyzed for all consecutive contractions in each rat (varying from one to five) and values were averaged for each animal. EMG intensity during the VAR was assessed by integrating the EMG waveform over time. The change of EMG integral values during voiding activity was divided by the change during the same time interval pre-void, and represents the voiding-associated intensity of the rectus abdominis muscle EMG response [9]. The use of the ratio rather than subtraction of baseline from the voiding-associated levels normalizes interanimal baseline variations. Intercontraction interval (ICI) was calculated by dividing the time from the initiation of the first void to the initiation of the last void by the number of voids. Blood pressure response to voiding activity (VVR) was calculated by subtracting the average of the pre- and post-void pressures from the peak pressure during voiding. Average per-void volume (VV) was calculated by dividing the total voided volume by the number of voids. Average flow rate (Q_ave_) was calculated by dividing per-void volume by time for each void and taking the mean of these values. Mean values of the parameters for each animal were averaged within each group and the groups were compared using the Student t test, P < 0.05 considered significant. Mean values and standard errors of measurement (SEM) were tabulated, and results in the text are given as mean +/- SEM.

## Results

Table 1 presents the average values of the assessed parameters for each experimental group. As these animals voided in response to physiologic bladder filling, the data from the intact animals represent normal voiding parameters in urethane-anesthetized female rats.

Figure 1 demonstrates EMG and intraperitoneal pressure changes accompanying voiding in intact and botulinum toxin-A pretreated animals. A significant increase in rectus abdominis EMG activity during voiding in the intact animals was observed. Pretreatment of the rectus abdominis muscle with botulinum toxin-A eliminated EMG evidence of abdominal wall activity, and the EMG activity ratio was significantly less than in the intact free voiding group. A positive abdominal pressure deflection was observed to accompany voiding in the intact rat (total 3 voids), and there was neither visible evidence on the abdominal pressure tracing nor calculated pressure differences in any BTX void (total 13 voids) despite pressure deflections in response to manual squeezing. Per-void volume and average urine flow were significantly decreased by BTX pretreatment.

**Figure 2:**
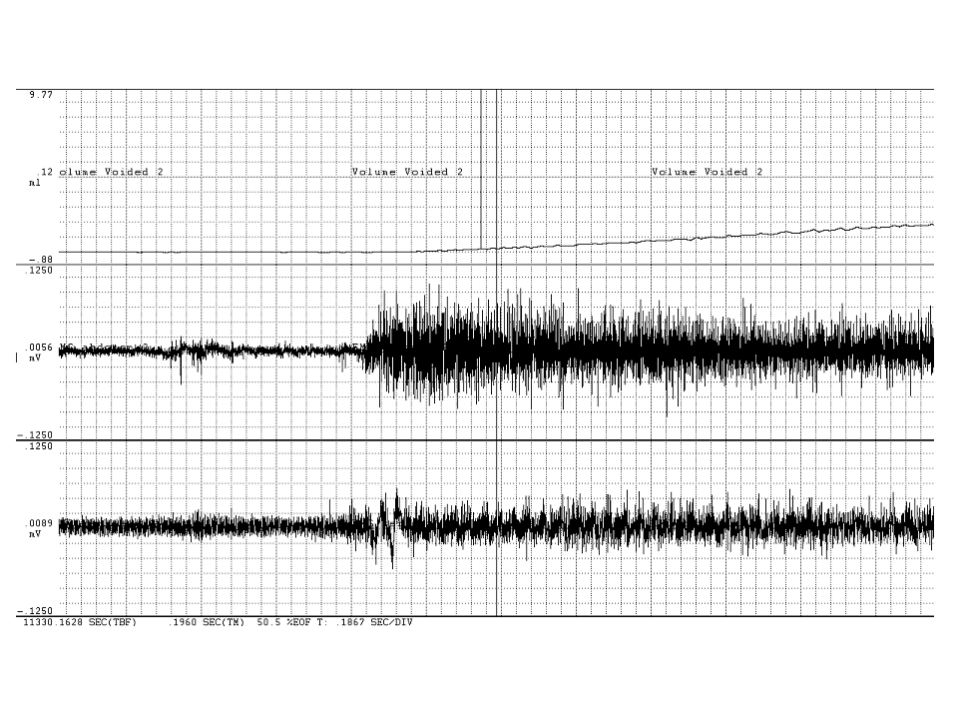
**Time-expansion of voiding-associated EMG changes**. Time-expanded tracings demonstrating changes in rectus abdominis and perineal EMG activity accompanying voiding. Top tracing is voided volume, middle tracing is rectus abdominis EMG, and bottom tracing is perineal EMG tracing. Pulse width of perineal EMG tracing indicated by vertical lines, interval is 0.2 second.

Perineal EMG intensity level was variable among rats during voiding, with the average integrated value slightly less than unity, and did not differ between groups. While not subjected to more detailed analysis, the waveform of the perineal EMG signal changed during urine flow from a tonic discharge to bursting activity occurring at approximately 0.2 second intervals as shown in Figure 2, in both groups of rats. ICI, blood pressure, and VVR were unchanged following BTX pre-treatment.

## Discussion

This study demonstrates that intra-abdominal pressurization due to the voiding-associated abdominal wall response is a normal and necessary component of voiding in urethane-anesthetized female rats. While the voiding-associated EMG response appears to be similar to a VMR, several differences exist. The abdominal wall activation during voiding is a physiologic response, in contrast to the VMR which by definition is a response to noxious stimulus. We have observed the voiding-associated response to be associated with urine flow rather than bladder pressurization during transurethral cystometry [2], whereas the VMR is reported to be a response to sudden bladder pressurization [3,9].

The increase of abdominal wall EMG intensity during voiding suggests that the voiding-associated abdominal wall contraction produces an increment of intra-abdominal pressure which contributes to the total bladder pressure available to drive urine flow. Cruz et al. demonstrated abdominal wall EMG activation in several points in the abdominal wall during voiding, including the oblique muscle groups and the rectus abdominis [1]. Logic dictates that such universal abdominal wall EMG activity signifying muscle contraction will be accompanied by an elevation of intra-abdominal pressure, and this has been observed [1]. Botulinum toxin eliminates neuromuscular signalling, thus the absence of a voiding-associated EMG response in the BTX-prepared rats signifies a lack of voiding-associated contraction of the rectus abdominis. This longitudinal segment of the abdominal wall thus remains compliant and therefore prevents (or at least minimizes) pressurization resulting from the remaining abdominis oblique contraction. Our methodology sought only to qualitatively evaluate the described voiding-associated abdominal pressure wave and its elimination by BTX preparation. The positive pressure increase observed in all three tested rats in response to abdominal squeeze confirms the ability of the technique to detect the presence of a pressure increase accompanying EMG activity, as a failure of the method (for example, an insufficient fluid column would place the catheter tip against a viscus surface) would eliminate the ability to detect a pressure change. The lack of degradation of hemodynamic parameters in the BTX-prepared animals is consistent with the observed changes in urine flow rate and per-void volume being due to the lack of abdominal pressurization rather than systemic toxicity related to botulinum toxin.

The perineal electrodes were not specifically placed into the rhabdosphincter, in order to minimize experimental impact on lower urinary tract afferent signalling. The lack of enhanced perineal muscle EMG activity during voiding may signify a lack of specific muscle activation during voiding as might be expected in the rhabdosphincter, although the change in waveform to a bursting activity with a frequency of approximately 5 Hz is consistent with IPHFO activity. This is consistent with voiding-associated perineal muscle activity observed previously [7]. The slight decrease in voiding-associated EMG activity observed in the BTX prepared animals may be artifactual, representing a diminished signal intensity as the EMG electrodes were slightly pulled apart as the intact abdominal wall muscles contracted, or be the result of the altered waveform during voiding signifying a qualitative change in muscle activity rather than an quantitative increase in muscle activation.

Bladder pressurization against intermittent urethral resistance appears to be essential for efficient voiding in the rat. Rhabdosphincter EMG activity increases during bladder filling [5]. The tonic resistance to urine flow against bladder pressurization persists until the intermittent pulsatile high frequency oscillatory (IPHFO) activity of the rhabdosphincter commences after initial bladder pressurization during a void [4]. In female rats, urine flow is intermittently reduced (but not stopped) by the IPHFO contractions, producing momentary increases in bladder pressure and decreases in urethral pressure distal to the EUS [4]. Elimination of this pressure differential at the EUS by proximal urethral restriction by ligature impairs voiding [10], suggesting that transmission of bladder pressure to the proximal urethra is important for the generation of urine flow. Elimination of rhabdosphincter function via lumbosacral motor nerve transection decreases voiding efficiency, an effect more pronounced in male rats (45% decrease) than in female rats (24% decrease) [5]. Intravenous d-tubocurarine also impairs voiding efficiency, and this effect has been ascribed solely to the resultant paralysis of rhabdosphincter and therefore elimination of the pulsatile phase of voiding [7,11], even though the additional paralysis of the abdominal wall may have contributed to these findings. All these findings suggest that normal voiding requires that the EUS generate resistance to urine flow and thus allow pressurization of the more proximal urethra and bladder.

The bladder may be pressurized directly by detrusor contraction, or indirectly by transmitted abdominal pressure. As the bladder fills, increasing activity on pelvic afferents in response to increasing wall stress triggers efferent firing with resultant detrusor contraction, and bladder pressure begins to rise [12,13]. Shortly thereafter, the abdominal wall is activated and concurrently the IPHFO/flow phase begins, possibly in response to urine being forced into the proximal urethra at a threshold bladder pressure against the closed rhabdosphincter. This timing suggests that abdominal pressure due to the voiding-associated abdominal response may thus play a part in initiating and supporting the IPHFO/flow phase of micturition.

It has been demonstrated that the voiding-associated EMG activity depends upon pelvic nerve afferents [1]. Bladder pelvic nerve activity decreases during the IPHFO phase, possibly in response to decreasing volume, with a change in efferent activity to a bursting pattern [14], and thus detrusor-generated bladder pressure may decrease. The persistence of the abdominal activity throughout the IPHFO/flow phase despite decreasing bladder afferent activity is consistent with afferent signalling leading to the abdominal response arising in the urethra rather than the bladder. The voiding-associated abdominal wall response may therefore provide an important self-sustaining quantum of driving pressure at a moment when detrusor-generated pressure is declining, and thus maintain urine flow during EUS relaxation. According to this hypothesis, failure of abdominal pressurization (as in the BTX group) results in a degradation of the IPHFO phase as the bladder empties, decreasing per-void volume and impairing flow. Our findings are consistent with such a functional role of abdominal wall contraction as part of normal voiding. Removing the supplemental pressure supplied by the abdominal wall without disturbing the rhabdosphincter activity decreased voiding efficiency as evidenced by a decreased per-void volume and flow rate.

The observed responses in this study of urethane anesthetized rats should be fundamentally the same as in non-anesthetized rats. Urethane anesthesia has minimal effect on afferent and efferent functions, has little influence on neurotransmitter release, slightly depresses detrusor responsiveness, and preserves voiding reflexes at cystometry [6,15-18]. However, under cystometric conditions, the bladder capacity may be decreased by urethane [18], although in rats with chronic suprapubic catheters, no decrement in capacity was attributed to urethane anesthesia [19]. We observed a larger average void (1.8 +/- 0.2 ml) occurring at a longer average interval (60.0 +/- 14.6 minutes) in intact anesthetized rats than reported in awake rats. Cruz et al. [1] reported a voiding volume and interval of 1.0 +/- 0.08 ml and 41 +/- 1 minutes in awake, free voiding (but partially restrained) rats. Our observed per-void volume is consistent with both an increased diuresis due to the relatively rapid infusion rate and the longer ICI. The difference in ICI between our study and that of Cruz et al. may be the combined result of the methodologic differences between the two studies. Rapid bladder filling results in delayed afferent signalling [20]. The enhanced diuresis due to vigorous hydration in our study may have prolonged the ICI relative to that resulting from typical bladder filling rates in unrestrained/unanesthetized rats. The ICI may have been further shortened in the previous study by anxiety produced by restraint in awake rats. The discrepancy between volume infused (4.8 ml/hr infused vs. 1.8 ml/hr voided) represents volume loading. Over the course of a 4–6 hour long observation period, a volume surplus certainly developed of a magnitude similar to Davis' acute volume loading [9]. Their observation was that GFR increased to accommodate this load, and therefore had our rats survived post-experiment, this excess volume (minus insensible and metabolic losses) would have been excreted. It seems unlikely that this aggressive hydration could alter the abdominal wall response to voiding or the urine flow parameters resulting from physiologic diuresis.

## Conclusion

Voiding-associated abdominal wall contraction is a normal and necessary component of voiding in urethane anesthetized rats. The proximal urethra may be the origin of the afferent signalling which results in the voiding-associated abdominal wall response. The importance of the bladder pressure increment due to the voiding-associated abdominal wall contraction may be in maintaining a normal duration intermittent pulsatile high frequency oscillatory (IPHFO)/flow phase and thus efficient voiding. In order to distinguish this physiologic somatic response to a normal visceral stimulus from the visceromotor response (VMR) elicited by noxious visceral stimuli, we suggest terming this aspect of voiding the Voiding-associated Abdominal Response (VAR).

## Competing interests

CPS is an Investigator for Allergan, Inc.

## Authors' contributions

PPS designed and conducted the experiments, and drafted the manuscript and its revisions

CPS and TBB provided guidance, oversight, and critical review of the manuscript

GTS aided in the design of the experiment and statistical analysis, provided critical oversight of the experiment, and provided critical review of the manuscript.

All authors have read and approved the submitted manuscript

## Pre-publication history

The pre-publication history for this paper can be accessed here:



## Figures and Tables

**Table 1:** Voiding-associated parameters in intact and botulinum-toxin prepared free voiding rats.

**Group**	n		**EMG abd**	**EMG peri**	**ICI**	**VV**	**Qave**	**BP**	**VVR**
			
			(ratio)	(ratio)	min	ml	ml/sec	mmHg	mmHg
**FV**	6	mean	**1.437**	**1.041**	**60.0**	**1.8**	**0.17**	**131**	**1.5**
		SEM	0.167	0.102	14.6	0.2	0.03	12	7.7
**FV+BTX**	8	mean	**0.936**	**1.095**	**59.0**	**1.1**	**0.11**	**151**	**2.7**
		SEM	0.041	0.148	18.0	0.2	0.02	7.3	1.5
		***p***	***0.003***	*n.s.*	*n.s.*	***0.036***	***0.048***	*n.s.*	*n.s.*

FV – intact free voiding rats. FV+BTX – botulinum toxin prepared rats. EMG abd – ratio of rectus abdominis voiding-associated EMG intensity to baseline. EMG peri – ratio of perineal voiding-associated EMG intensity to baseline. ICI – intercontraction interval (minutes). VV – per-void volume (ml). Qave – average voiding urine flow rate (ml/sec). BP – blood pressure. VVR – voiding-associated viscerovascular reflex. Values are means, with standard error of measurement (SEM). Statistical comparisons made by t-test, *p *< 0.05 significant.
